# Performance Degradation of a Double-Perovskite PrBaCo_2_O_5+δ_ Cathode Operating under a CO_2_/H_2_O-Containing Atmosphere

**DOI:** 10.3390/molecules29051063

**Published:** 2024-02-29

**Authors:** Lin Zhu, Pengzhang Li, Yuanyuan Li, Xiaonan Fu, Yuanyuan Qi, Juntao Wang, Zaixu Liu, Hongyan Yang

**Affiliations:** 1 School of Sciences, Henan University of Technology, Zhengzhou 450001, China; 2 Institute of New Energy Materials and Devices, School of Materials Science and Engineering, Jingdezhen Ceramic University, Jingdezhen 333403, China

**Keywords:** PrBaCo_2_O_5+δ_, BaO, segregation, grain boundaries

## Abstract

The electrochemical activity and stability of the PBCO electrode are investigated under the annealing processes in an atmosphere containing CO_2_/H_2_O for solid oxide fuel cells (SOFCs). The electrochemical impedance spectrum results unequivocally confirm the significant deterioration in PBCO cathode performance upon annealing under ambient air conditions, particularly when exposed to CO_2_/H_2_O atmospheres. Microstructure and surface chemical state analyses reveal the segregation of BaO on the PBCO surface, and the formation of insulating BaCO_3_ degraded the electrochemical performance. CO_2_ and H_2_O exhibit a significant induced effect on the segregation of Ba in PBCO to the surfaces, thereby causing a rapid decline in electrode performance. Additionally, the analysis of volume relaxation reveals that the presence of oxygen in the electrode environment can also influence the deposition process occurring on the surface of the electrode. However, this phenomenon is not observed in N_2_. This study emphasizes the impact of various gases present in the working atmosphere on surface-separated BaO, which consequently plays a pivotal role in the activity and long-term stability of PBCO electrodes.

## 1. Introduction

The SOFCs exhibit a remarkable energy conversion efficiency, minimal emission of pollutants, and the ability to utilize a diverse range of compatible fuels, which contribute significantly to their notable advantages [1,2]. The cathode, being one of the pivotal components in RSOFCs, primarily facilitates the process of oxygen reduction (ORR) [3,4,5]. The ORR at the cathode involves the adsorption and subsequent desorption of oxygen molecules on its surface, as well as surface diffusion and charge transfer through ionization [6]. Due to considerations related to battery commercialization, air is typically chosen as the working atmosphere for the cathode. Consequently, the electrochemical activity and stability of the cathode in an air environment are crucial factors that impact RSOFC performance and operational lifespan.

Traditional La_1−x_Sr_x_MnO_3_ (LSM) cathodes primarily limit the ORR to the three-phase boundary (TPB), where they interface with the electrolyte and air, due to their limited ion conductivity. By substituting traditional cathodes with single-phase mixed ionic–electronic conductor (MIEC) materials, it becomes possible to expand the active area of the electrode surface, thereby enhancing ORR performance. Consequently, MIEC materials such as Sm_1−x_Sr_x_CoO_3_ [7], La_0.6_Sr_0.4_Co_0.2_Fe_0.8_O_3_ [8], and Ba_0.5_Sr_0.5_Co_0.8_Fe_0.2_O_3−δ_ [9] have been developed. Perovskite oxides represent one type of the most promising candidates for use as the cathode in intermediate temperature SOFCs and many new materials have been reported to show outstanding ORR activity [10,11]. Especially, the double perovskite PrBaCo_2_O_5+δ_ (PBCO), characterized by mixed ionic–electronic conductivities, has been proposed to exhibit the highest ORR performance among the LnBaCo_2_O_5+δ_ (Ln = La, Pr, Nd, Sm, Gd, and Y) family due to its abundant oxygen vacancies and anisotropic oxygen ion mobility [12,13,14,15,16,17,18]. The study conducted by Burriel et al. [19] revealed that, at a temperature of 700 °C, the oxygen diffusion coefficient and surface exchange coefficient of PBCO were significantly higher (approximately 7 and 3 orders of magnitude, respectively) compared to LSM under similar conditions [20]. The measured values for PBCO were approximately 10^−8^ cm^2^s^−1^ and 10^−6^ cms^−1^, respectively. According to a study conducted by Zhang et al. [21], the comparative analysis of layered oxides reveals that PBCO exhibits the lowest polarization resistance at 600 °C, measured as 0.213 Ω cm^2^. Additionally, PBCO demonstrates an electrical conductivity of approximately 800 Scm^−1^ at 800 °C, which is significantly higher than that of LSM (~100 Scm^−1^) [22]. However, Ba precipitation occurs on the surface of the PBCO electrode, leading to the degradation of electrode performance [23]. According to Druce et al., elemental Ba is observed to precipitate outward from the lattice, with Ba enrichment occurring at temperatures as low as 400 °C [17,24,25]. Insulating oxides on the cathode surface hinder active ORR sites and impede oxygen reduction and transport kinetics. In solid oxide fuel cells, air is typically chosen as the working atmosphere, containing various gases such as N_2_, O_2_, CO_2_, and H_2_O, etc. Further investigation is warranted to understand the impact of these gases on the activity and stability of the cathode.

The present study investigates the impact of the annealing atmosphere on the electrochemical activity and stability of the PBCO cathode. The findings reveal a close correlation between electrode activity, microstructure, and chemistry. Notably, the formation of insulating BaCO_3_ nanoparticles on the surface of PBCO is identified as the primary factor contributing to the decline in electrode activity. Furthermore, this paper discusses the underlying mechanism involving BaO species when subjected to annealing in air, CO_2_, and H_2_O.

## 2. Results and Discussion

### 2.1. Electrochemical Performance

The working atmosphere of the RSOFC oxygen electrode is usually selected as air, which typically contains around 3% H_2_O and 0.04% CO_2_; both of these components have the potential to affect the stability of the electrode. Hence, we performed electrochemical performance tests on the electrode under these concentrations (air, 3% H_2_O–air) and their multiples (4%, 8%, and 80% CO_2_–air).

**Figure 1:**
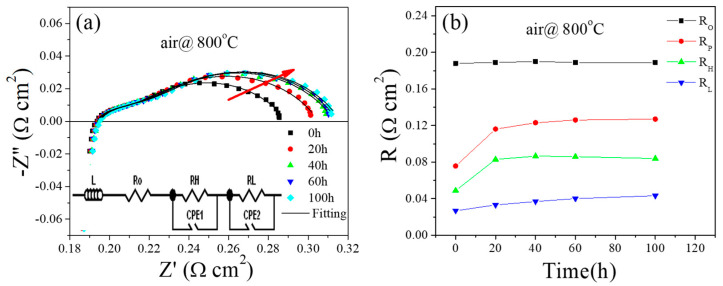
(**a**) Electrochemical impedance spectra of a PBCO cathode annealing at 800 °C for 100 h in air. The illustration in (**a**) is an equivalent circuit (L is inductance, RO is the ohmic resistance, CPE is the constant phase element, and (RH, CPEH) and (RL, CPEL) represent the high-frequency arc and low-frequency arc, respectively) for impedance data fitting; (**b**) corresponding fitting results of Rp (RH + RL) as a function of time.

The electrochemical impedance spectra of the annealed PBCO cathode were tested at 800 °C for 100 h under open-circuit conditions in order to investigate the impact of air annealing on electrode activity (Figure 1). After the sintering process, the ohmic resistances (Ro) exhibit a slight decrease with processing time, which can be attributed to the further combination between the silver current collector and electrode. However, the electrode polarization resistance (Rp) increases from an initial value of 0.08 to 0.12 Ω cm^2^ (an increase of approximately 50%) after undergoing a heat treatment for 100 h. Nevertheless, during the initial testing period (~40 h), there is a decrease in the performance of the PBCO electrode in flowing air, followed by a stable state. The impedance responses were segregated into two electrode processes at high and low frequencies. The low-frequency arc is potentially ascribed to the adsorption and diffusion step in the gas phase, while the high-frequency arc corresponds to electron charge transfer at the TPB [26]. With the prolonged annealing time, a noticeable enhancement in RL associated with low-impedance arcs was observed, indicating the suppression of gas-phase adsorption and the diffusion step in oxygen reduction reaction kinetics. This finding is consistent with Wachsman’s study on the surface elemental enrichment behavior of La_0.6_Sr_0.4_Co_0.2_Fe_0.8_O_3_ through heat treatment [27]. They discovered that Sr segregation can significantly deteriorate the catalytic activity of LSCF’s surface. Moreover, it has been reported that Sr segregation depresses the surface exchange kinetics of LSM films due to the insulating nature of SrO species [28]. Additionally, thermal sintering leads to grain growth and agglomeration in electrodes, resulting in a decrease in TPB length and consequently an increase in Rp.

**Figure 2:**
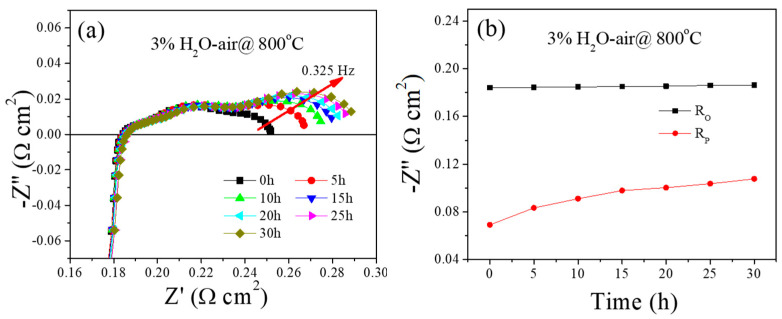
Electrochemical impedance spectra of PBCO electrode annealed at 800 °C for 30 h. (**a**) 3% H_2_O–air, (**b**) the corresponding fitting results of Rp as a function of time.

The impact of H_2_O impurity on PBCO electrode activity was investigated by subjecting it to a calcination process at 800 °C for 30 h in an atmosphere containing 3% H_2_O (Figure 2). After operating for 30 h, the Ro value remained relatively stable throughout the entire duration of the test, while Rp significantly increased from 0.07 to 0.11 Ω cm^2^. Additionally, no intersection was observed between the substantial impedance arc of the PBCO electrode and the Zreal axis in a H_2_O–air environment. The impedance spectra of the PBCO electrode exhibit three distinct arcs, indicating the involvement of at least three rate-limiting steps. Notably, a significant change is observed in the low-frequency arc (such as a frequency of 0.325 Hz) with annealing time. These findings suggest that the gas diffusion process may serve as the rate-limiting step. The limitation of gas diffusion can arise from either bulk gas diffusion within the porous structure of the cathode or the surface diffusion of oxygen on the cathode surface.

The research conducted by Mojie Cheng et al. has revealed that the presence of both CO_2_ and H_2_O in the gas phase facilitates the occupation of oxygen vacancies in metal oxides due to strong co-adsorption [29]. The existence of an oxygen vacancy in a perovskite-type cathode is crucial for oxygen reduction. These findings imply that the significant resistance to gas diffusion observed in the PBCO electrode operating under 3% H_2_O–air flow is not attributed to bulk gas diffusion, but rather to the surface diffusion of oxygen on the PBCO surface. Operating the PBCO electrode at 800 °C in a 3% H_2_O–air environment may induce changes in its surface morphology.

**Figure 3:**
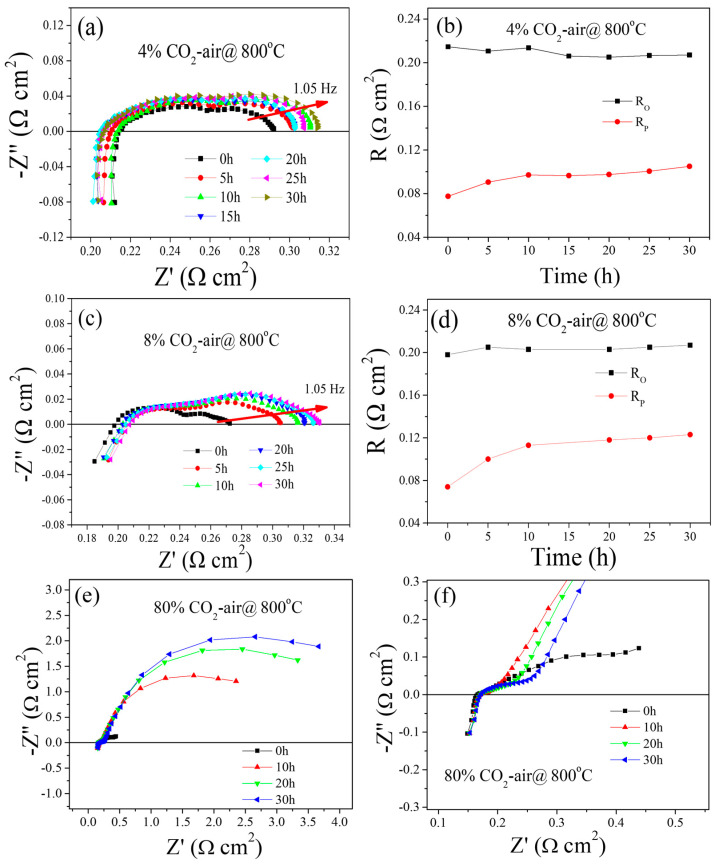
Electrochemical impedance spectra of a PBCO electrode annealed at 800 °C for 30 h and the corresponding fitting results of Rp as a function of time. (**a**,**b**) 4% CO_2_–air, (**c**,**d**) 8% CO_2_–air and (**e**,**f**) 80% CO_2_–air.

The effects of CO_2_ impurities on electrode properties were tested using three different concentrations of CO_2_. The impedance spectra of the PBCO electrode were annealed in air with 4%, 8%, and 80% CO_2_ at a temperature of 800 °C for a duration of 30 h (Figure 3). 

All results indicate that Ro remains relatively unchanged, while the Rp gradually increases during the entire duration of the test. Figure 3a,b illustrates the impact of 4% CO_2_ on the electrochemical performance of PBCO cathodes. The initial Rp value for the original PBCO electrode is measured at 0.08 Ω cm^2^, which then increases to 0.11 Ω cm^2^ (an increase of approximately 38%). With an increase in CO_2_ concentration to 8%, a significant rise the in initial Rp from 0.08 to as high as 0.13 Ω cm^2^ was observed, resulting in a total increment of approximately 63%. Figure 3b,d represent similar trends between these two groups of data, where Rp exhibits gradual growth within the first ten hours followed by relative stability throughout the remaining testing period. When the CO_2_ concentration rises to 80%, Rp exhibits rapid growth throughout the entire annealing process (Figure 3e,f). Comparing these results, it is evident that the degradation becomes more severe with an increased CO_2_ concentration for the PBCO electrode. It should be noted that in these cases, the increase in Rp is primarily attributed to changes in the low-frequency arc (such as a frequency of 1.05 Hz), which are associated with surface oxygen adsorption and diffusion processes. This indicates that CO_2_ impurities can hinder ORR and lead to a rapid deactivation of PBCO electrodes [23].

### 2.2. Structure Characterization

**Figure 4:**
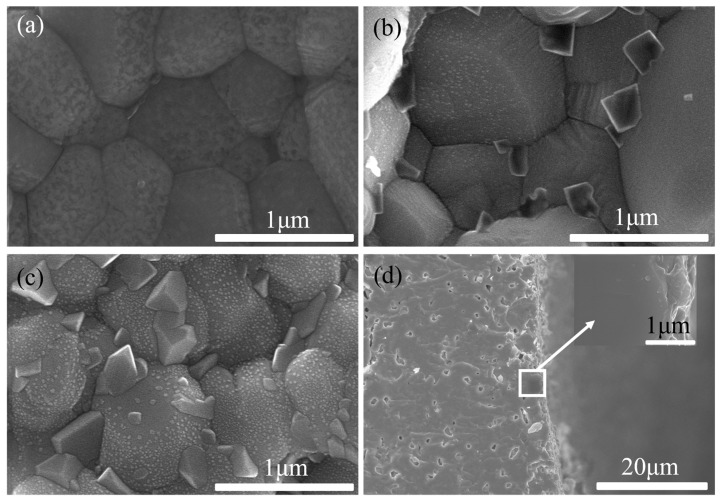
The SEM images of the surface of a PBCO plate annealing in air at 800 °C, (**a**) As-prepared, (**b**) annealed 30 h, (**c**) annealed 100 h, (**d**) the cross section of (**c**).

The SEM micrographs in Figure 4 depict the surfaces of the PBCO pellets before and after being annealed at 800 °C in air. For the initial electrode, PBCO particles have an approximate particle size of 0.5 μm and exhibit dense and smooth surfaces, as depicted in Figure 4a. However, after undergoing 30 h of annealing, the PBCO particles experience coarsening, and a few uniform nanoparticles (~15 nm) emerge on the previously smooth grain surfaces. Interestingly, larger polygonal precipitates are formed along the grain boundaries (refer to Figure 4b). With an extended treatment time of 100 h, more noticeably larger particles (~33 nm) can be observed. The presence of these precipitates became more prominent and exhibited a higher areal density as the annealed time increased (Figure 4c). 

The precipitates typically manifested themselves in the form of spheres and polygons. Specifically, a spherical type was observed exclusively on the surfaces of PBCO grains, while larger polygonal precipitates formed solely along grain boundaries. These observations suggest that an incoherent curved interface demonstrates a rapid growth rate, whereas a semicoherent facet exhibits slower growth. The grain boundary is the region where individual crystal units meet, characterized by a high defect density. Lattice shrinkage at the grain boundaries poses a greater risk to the original structure compared to surface damage. David N. Mueller reported that metallic element diffusion rates at grain boundaries are approximately three orders of magnitude higher than those observed in semicoherent facets [30]. This discrepancy in diffusion rates leads to the selective growth of precipitates. The cross-sectional image of an annealed sample exhibits exceptional density without any interconnecting holes (Figure 4d). Additionally, an inserted SEM image showcases a higher-magnification view of the cross-section’s edge. Notably, precipitates exclusively appear on the surface of the sample in direct contact with atmospheric conditions. The above results suggest that the Rp increases over time, which is consistent with SEM (Figure 4) studies. Van Der Heide reported that Sr segregation resulted from decreased stability at the surface and structural distortion due to the abrupt termination of the lattice structure [31].

**Figure 5:**
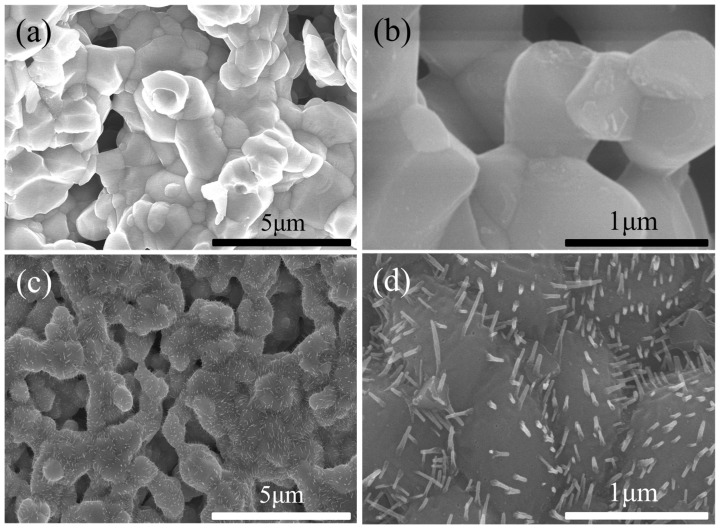
The SEM images of a PBCO electrode. (**a**,**b**) As-prepared, (**c**,**d**) annealed in 3% H_2_O–air at 800 °C for 30 h.

The SEM images of the PBCO electrode before and after operating under 3% H_2_O–air at 800 °C for 30 h are presented in Figure 5. As shown in Figure 5a,b, the initial electrode exhibits porous agglomerates consisting of smooth and pristine surfaces of PBCO particles. Following 30 h of operation with 3% H_2_O, a porous electrode structure that facilitates gas bulk diffusion is maintained, but additional acicular segregation emerges on the surface of PBCO particles (see Figure 5c,d), significantly altering the microstructure of the electrode. The presence of an aqueous air atmosphere triggers accelerated segregation on the electrode surface, resulting in a decrease in its activity. The findings from the SEM test corroborate those obtained from the impedance spectrum test illustrated in Figure 2.

**Figure 6:**
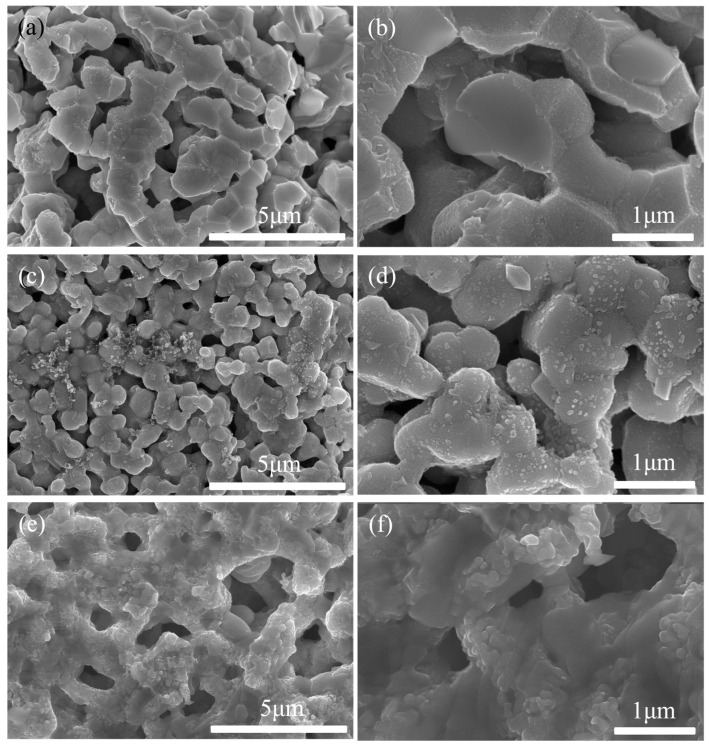
The SEM images of a PBCO electrode annealed at 800 °C for 30 h. (**a**,**b**) 4% CO_2_–air, (**c**,**d**) 8% CO_2_–air, (**e**,**f**) 80% CO_2_–air.

The SEM images of PBCO electrodes annealed at 800 °C for 30 h in atmospheres containing 4%, 8%, and 80% CO_2_ are presented in Figure 6a–f. After annealing in a 4% CO_2_–air atmosphere, the surface of the PBCO grains exhibited roughness, suggesting that surface segregation may have occurred during the process. Numerous particles emerged on the surface of PBCO grains, with sizes ranging from 19 to 76 nm (Figure 6a,b). With an increase in CO_2_ concentration to 8%, a higher quantity and larger size of particles were observed on the surface of the PBCO grains (Figure 6c,d). The average particle size ranged from 33 to 140 nm. Subsequent heating in an atmosphere containing 80% CO_2_ led to an enlargement of most particles to approximately 200 nm, with distinct fuzzy boundaries visible on the surface of PBCO (Figure 6e,f). The results suggest that elevated levels of CO_2_ in the air facilitate the segregation and formation of these nanoparticles. Consequently, these particles obstruct the active sites responsible for oxygen reduction reactions on the electrode, leading to a decline in its performance, which is consistent with the observed increase in Rp as shown in Figure 3.

### 2.3. Discussion

**Figure 7:**
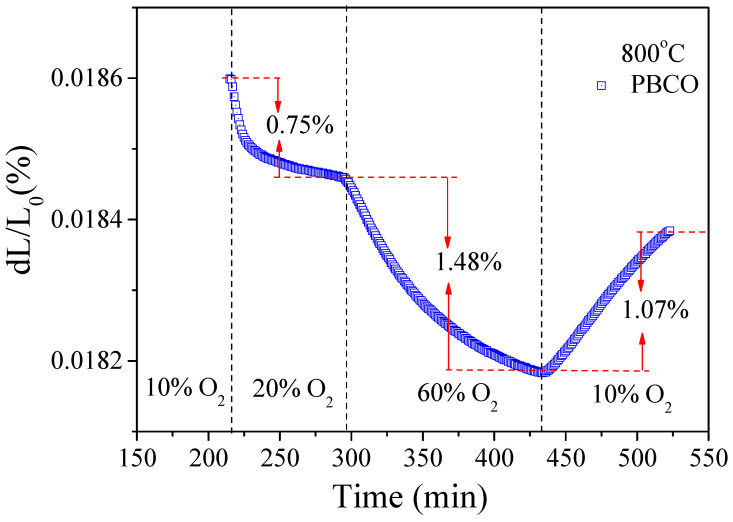
Volume relaxation of a dense PBCO bar under the different *P*O_2_ at 800 °C.

The cathode plays a pivotal role in RSOFC by facilitating the reduction of oxygen. We conducted further analysis on the size relaxation of dense PBCO bars at 800 °C under varying levels of *P*O_2_ (controlled by the argon–oxygen ratio). Our results indicate that an increase in *P*O_2_ (from 0.1 atm to 0.6 atm) is accompanied by a distinct volume shrinkage and intensification of lattice contraction, while a decrease in *P*O_2_ (from 0.6 atm to 0.1 atm) leads to lattice expansion (see Figure 7). The alteration of *P*O_2_ directly influences the lattice contraction/expansion of PBCO, which is closely associated with variations in oxygen content and the subsequent oxidation/reduction of Co ions. This results in a change in their radius, with the ionic radii for Co^4+^ and Co^3+^ measuring 0.067 and 0.0685 nm, respectively, when in a high spin state with six-coordinate geometry. The reduction in size observed in PBCO samples can be attributed to lattice shrinkage under high oxygen partial pressure due to excessive oxygen incorporation and the consequent oxidation of Co ions at PBCO lattice sites, leading to the additional outcome of surface Barium precipitation [25]. The segregation behavior in LSCF and LSM has been previously suggested to be primarily influenced by variations in the oxygen environment, resulting in chemical expansion [27,28].

**Figure 8:**
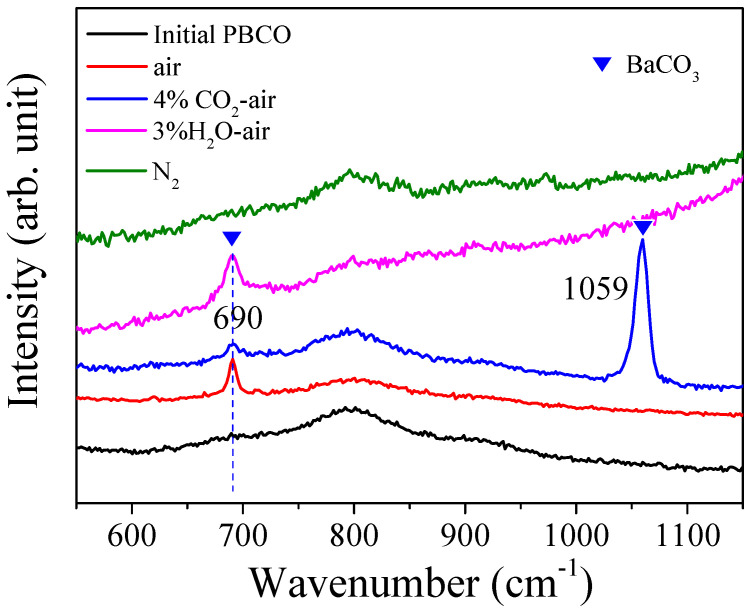
Raman spectra of initial and annealed PBCO obtained at 800 °C for 30 h under different atmospheric conditions.

The presence of the surface segregation phase was further confirmed through analysis of the Raman spectra obtained from examining the oxygen electrode in PBCO before and after undergoing annealing at a temperature of 800 °C for a duration of 30 h under various atmospheric conditions (as shown in Figure 8). The Raman spectroscopy analysis was performed at room temperature. Notably, distinctive peaks corresponding to BaCO_3_ were observed upon annealing in an air environment, with one peak specifically identified at 689 cm^−1^ representing the doubly degenerate bending mode and symmetric stretching vibration associated with CO_3_^2−^ ions [32], recognized as key vibrational modes for BaCO_3_. The formation of BaCO_3_ is likely attributed to the reaction between BaO and CO_2_ present in the flowing air. Upon cooling to room temperature under an argon atmosphere, the annealed PBCO sample confirms the generation of BaCO_3_ during the annealing process. Mahapatra’s findings indicate that the interaction between SrO and CO_2_ on the LSM surface leads to the formation of SrCO_3_. The presence of SrCO_3_ in the as-fabricated LSM cathodes suggests that its formation is driven by thermodynamics [6]. After annealing in 4% CO_2_–air, the emergence of vibration peaks at 690 and 1059 cm^−1^ indicates the probable formation of a significant quantity of BaCO_3_ during the annealing process (Figure 3 and Figure 6). The presence of CO_2_ tends to react with BaO on the surface of PBCO, resulting in the generation of BaCO_3_ which obstructs oxygen adsorption sites. Consequently, there is a rapid deterioration in electrochemical performance [6,23,25]. After exposure to a 3% H_2_O–air environment, the Raman spectrum of the electrode shows the characteristic vibration peak of BaCO_3_. However, there are no significant changes observed in the Raman spectrum after annealing in N_2_ gas. These findings indicate that CO_2_ and H_2_O have a significant impact on inducing the surface segregation of Ba within PBCO.

In this study, a significant decline in the performance of PBCO electrodes was observed under conditions containing CO_2_, accompanied by notable changes in microstructure. Based on our experimental observations, we propose a schematic representation of the underlying mechanism for the degradation of PBCO cathodes during testing. Initially, the annealing process leads to the surface segregation of barium on PBCO and the simultaneous formation of cation vacancies. The segregated Ba element predominantly exists as BaO according to Equation (1). In the presence of CO_2_, interaction between BaO and CO_2_ results in the formation of BaCO_3_ according to Equation (2).

(1) $$Ba_{\mathrm{Pr}}^{\prime }+\frac{1}{2}O_{2}(g)\to BaO(s)+V_{\mathrm{Pr}}^{\prime \prime \prime }+2h^{•}$$

(2) $$BaO(s)+CO_{2}(g)↔BaCO_{3}(s)$$

The results from Figure 2 and Figure 5 demonstrate that the presence of H_2_O exacerbates the chemical reaction between CO_2_ and PBCO, leading to the formation of Ba(OH)_2_ species. This phenomenon is particularly pronounced at the cathode/electrolyte interface where a significant number of oxygen vacancies exist. Equation (3) represents this reaction, while Equation (4) illustrates how the resulting Ba(OH)_2_ readily migrates on surfaces and reacts with CO_2_ to produce BaCO_3_.

(3) $$\mathrm{Pr}BaCo_{2}O_{5+\delta }+xH_{2}O↔\mathrm{Pr}Ba_{1-x}Co_{2}O_{5+\delta -x}+xBa{\left(OH\right)}_{2}$$

(4) $$Ba{\left(OH\right)}_{2}+CO_{2}↔BaCO_{3}+H_{2}O$$

**Figure 9:**
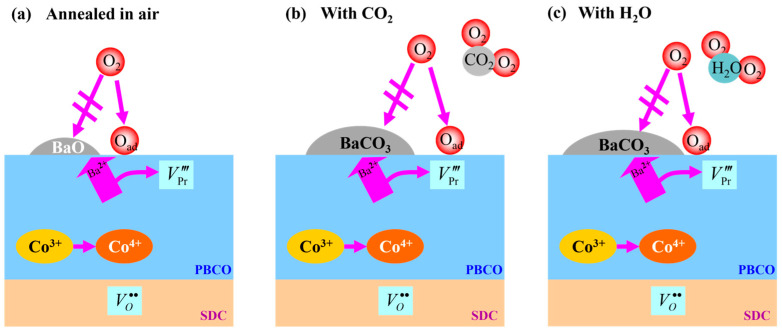
An illustration depicting the potential mechanism of PBCO cathode’s interaction with H_2_O and CO_2_ is presented. (**a**) The PBCO cathode undergoes high-temperature sintering in an air environment, resulting in the formation of BaO species. (**b**) Annealing in a CO_2_–air atmosphere. (**c**) Annealing in a H_2_O–air environment. Both H_2_O and CO_2_ exert a certain level of influence on the segregation of Ba on the electrode surface, leading to the formation and accumulation of an insulating phase known as BaCO_3_. This obstructs oxygen incorporation pathways and consequently results in performance degradation.

The phenomenon of cation segregation is believed to be driven by a combination of electrostatic and elastic interactions resulting from the mismatch in size and charge between the dopant and host ions [33]. This leads to an excess accumulation of enriched species on the electrode surface, which acts as a barrier inhibiting oxygen exchange reactions at the surface [34]. Previous studies have also indicated that Sr segregation on the surface contributes to the deterioration of oxygen reduction activities in (La,Sr)CoO_3_ and LSM materials [28,35]. Similarly, the presence of BaO species in the surface layer of PBCO electrodes, which acts as an insulator, can impede oxygen adsorption, diffusion, and surface exchange on the electrode surface by obstructing its path (Figure 9). This obstruction results in a significant initial loss in polarization for PBCO electrodes [23,25,35]. These findings suggest that subjecting the PBCO electrode to an annealing process in an atmosphere containing impurities may lead to decreased performance.

## 3. Materials and Methods

### 3.1. Fabrications

The Sm_0.2_Ce_0.8_O_1.9_ (SDC) powder was synthesized via a sol-gel process and subsequently uniaxially pressed into 13 mm-diameter discs, which were then sintered at 1400 °C for 4 h to obtain the electrolyte-support material [25]. PBCO powder was prepared using a modified sol-gel method, followed by the formation of a cathode slurry through mixing with ethyl cellulose and terpineol [25]. The cathode slurry was coated onto one side of the sintered SDC disc, which was further sintered at 1100 °C for 2 h in air to form the working electrode (WE). As for the counter electrode (CE), an Ag paste was symmetrically applied onto the opposite side of the SDC pellet. An Ag ring was brushed around the WE on the SDC pellet as the reference electrode (RE).

### 3.2. Characterization

The annealing process was periodically interrupted to conduct electrochemical impedance spectroscopy (EIS) measurements using a Bistat-Potentiostat (Bio-logic VSP) controlled by EC-Lab software (11.01). The EIS analysis of the half-cell (three-electrode) was performed under open-circuit conditions, with a frequency range of 0.1–10^6^ Hz and an AC signal amplitude of 10 mV. Impurity gas is mixed with air in terms of volume fraction, and the gas compositions are controlled by a mass flow controller (Seven Star Huachuang, Beijing, China). The steam concentration is regulated by the steam generator. The microstructures of the PBCO electrode before and after treatment were examined using a field emission scanning electron microscope (FE-SEM, Hitachi SU800, Hitachi, Tokyo, Japan). Raman spectrometry measurements were conducted using a Renishaw inVia system (WiRE^TM^ 2.0) with a 532 nm laser, in the wave number range of 200–1400 cm^−1^, to further investigate the surface segregation of the PBCO electrode. The in situ high-temperature structural evolution of dense PBCO bars was characterized by employing a thermal dilatometer (Netzsch DIL402/3/G, NETZSCH, Selb, Germany) at 800 °C.

## 4. Conclusions

The impact of an impurity atmosphere on the electrochemical performance, stability of microstructure, and chemical state of PBCO cathodes were investigated at a temperature of 800 °C. The presence of CO_2_ and H_2_O significantly influences the distribution of Ba on the surfaces in PBCO. The segregation or precipitation of BaO or Ba(OH)_2_ can readily react with CO_2_, leading to the subsequent formation of inactive BaCO_3_ on the surface of PBCO and consequent deterioration in its electrochemical performance. Only surface grains exposed to the gas exhibit Ba segregation, and their grain boundaries are more susceptible than the surface. A higher concentration of CO_2_ has a more adverse effect on PBCO performance due to the formation of a more prominent BaCO_3_ phase. The presence of N_2_ in the air does not result in Ba segregation, while an environment with a high oxygen partial pressure can promote the occurrence of BaO segregation due to lattice contraction in the PBCO electrode.

## Data Availability

Data are contained within the article.
